# Revamping Patient Transfers: A Comparative Assessment of Lean Readiness in Intensive Care and Postoperative Unit Admissions

**DOI:** 10.1155/jonm/1152328

**Published:** 2026-11-13

**Authors:** Sally Mohammed Farghaly Abdelaliem Abdelrahman, Mona Saad Abdelwareth Ahmed, Nesreen AbdelMonaem AbdelSataar AbouZeid

**Affiliations:** ^1^ Department of Nursing, Faculty of Allied Health Sciences Kuwait University Kuwait City Kuwait kuniv.edu; ^2^ Nursing Administration Department, Faculty of Nursing Alexandria University Alexandria Egypt alexu.edu.eg; ^3^ Critical Care and Emergency Nursing Department Faculty of Nursing Alexandria University Alexandria Egypt alexu.edu.eg; ^4^ Department of Medical and Surgical Nursing College of Nursing Princess Nourah bint Abdulrahman University P.O. Box 84428 Riyadh 11671 Saudi Arabia pnu.edu.sa; ^5^ Department of Medical Surgical Nursing Faculty of Nursing Alexandria University Alexandria Egypt alexu.edu.eg

**Keywords:** comparative study, general intensive care units, Lean assessment, Lean readiness, patient transfers, postoperative care units

## Abstract

**Background:**

Efficient patient transfers between the operating room (OR), postoperative care units (POCUs), and general intensive care units (GICUs) are vital for ensuring patient safety and quality of care. Traditional methods of patient transfer are often inefficient, creating delays and potential risks. Lean methodologies, which focus on reducing waste and improving process efficiency, may offer solutions, but little is known about how ready current patient transfer and admission processes are for Lean implementation.

**Aim:**

To assess the Lean readiness of patient admission processes in the GICU and POCU, focusing on the value‐added (VA) aspects of nursing practices, including time, cost, and effort during patient transfers.

**Method:**

A descriptive comparative design was applied to observe 100 nurses during 100 patient admissions in the GICU and POCU at a single general university hospital in Egypt. Using a Lean‐based observational checklist developed and psychometrically tested for this study, activities were timed, classified as VA or non‐value‐added (NVA), and compared between the two units. Lean was used as an assessment framework only; no Lean intervention was implemented.

**Results:**

Among 100 observed admissions (47 in GICU and 53 in POCU), the overall VA score was significantly higher in the GICU than that in the POCU (97.0 ± 2.4 vs. 95.9 ± 2.1; *p* = 0.037; effect size *r* = 0.21, small), with the largest difference for the use of a recognized assessment framework (VA score 89.6 ± 7.8 vs. 83.8 ± 2.4; *p* < 0.001; *r* = 0.41). Overall time, effort, and cost scores were also higher in the GICU, but the mean differences were small (2.1–4.6 points on a 0–100 scale). Effort for preparing to receive the patient differed significantly between units (*p* = 0.016; *r* = 0.24) although the means were similar (40.3 ± 6.0 in GICU vs. 39.6 ± 4.4 in POCU). Preparation for receiving patients ranked lowest on time, effort, and cost scores in both units. The average time spent per admission activity was 24.3 ± 21.2 min, with no significant difference between units (*p* = 0.528). These findings describe current admission practice and indicate where Lean‐based improvement could be targeted; they do not show any effect of Lean.

**Conclusion:**

Nursing practices during patient admissions differed modestly between the GICU and POCU: the GICU showed a higher VA contribution of structured assessment and higher overall time, effort, and cost scores, whereas the time in minutes was similar. Because no Lean intervention was implemented, the findings indicate partial Lean readiness and identify targets for future Lean‐based improvement, the effect of which still needs to be tested.

**Implications to Nursing Management:**

A Lean‐based assessment of admission workflows can help nurse managers identify NVA activities and set unit‐specific priorities for improvement; the effect of subsequent Lean implementation on safety and efficiency needs to be evaluated.

**Summary**
• What is known about this topic?
◦ Lean methodology aims to eliminate waste and improve efficiency in healthcare processes.
◦ It focuses on optimizing workflows, reducing non‐value‐added (NVA) activities, and enhancing performance.
◦ Lean techniques have been linked to improved process efficiency and reduced costs compared to traditional methods.
◦ Effective Lean implementation requires staff training, leadership support, and continuous improvement initiatives.
• What does this paper add?
◦ This study provides a comparative analysis of nursing activities during patient admissions in GICU and POCU, highlighting differences in time, effort, cost, and value‐added (VA) contributions.
◦ GICU nurses showed a higher VA score for structured assessment and higher overall time, effort, and cost scores than POCU nurses, while time in minutes was similar between units.
◦ Inefficiencies were identified in specific activities, such as preparation for receiving patients and reviewing patient notes, which are candidate targets for future Lean‐based improvement.
◦ Structured assessment frameworks, such as ABCDE, were associated with a higher VA score in the GICU although they involve higher resource use.
◦ The findings suggest a need for targeted staff training and unit‐specific workflow standardization; whether these reduce NVA activities without compromising patient outcomes has still to be tested.

## 1. Introduction

Patients admitted to general intensive care units (GICUs) represent a critically vulnerable population, typically facing unstable medical conditions that pose significant risks to their lives. These patients require immediate, coordinated interventions to address either actual or potential life‐threatening conditions. GICUs play a crucial role in modern healthcare systems, offering comprehensive life support interventions and intensive monitoring designed to stabilize patients, prevent further deterioration, and improve clinical outcomes [1, 2]. Common conditions leading to GICU admissions include sepsis, acute respiratory distress syndrome (ARDS), major trauma, and various other critical conditions that compromise vital organ functions [3]. These complex cases necessitate multifaceted approaches, including mechanical ventilation, hemodynamic monitoring, and renal replacement therapies [4]. Effective management in the GICU requires not only advanced technology but also the coordinated efforts of a multidisciplinary team, including intensivists, nurses, respiratory therapists, pharmacists, and other specialists [1, 5].

Continuous and meticulous monitoring in the GICU is paramount to detect early signs of clinical deterioration. Vital signs, such as the heart rate, blood pressure, oxygen saturation, and respiratory rate, must be constantly observed to ensure timely interventions [6]. This monitoring is a cornerstone of GICU care, allowing for the rapid adjustment of treatment strategies in response to the patient’s evolving condition [7]. GICU teams, through effective communication and coordination, play an essential role in the prompt detection and management of complications, thereby improving patient outcomes [8].

The period immediately following surgery is another critical phase for patients, particularly those emerging from surgery. In this vulnerable state, postoperative care units (POCUs) provide essential monitoring and care to ensure that patients safely recover from the effects of surgery [9]. The primary focus during this phase is maintaining vital physiological functions, including airway patency, ventilation, and circulation [10]. The transition from surgery to the POCU involves close observation of vital parameters, such as temperature, oxygen saturation, and pain levels, which are crucial for detecting potential complications and ensuring timely interventions [11]. In addition, pain management is a key concern in the postoperative period, as inadequate control of postoperative pain can delay recovery and negatively impact patient outcomes [12].

The role of postoperative nurses in the POCU is multifaceted, requiring them to monitor for signs of respiratory compromise, hemodynamic instability, and other postoperative complications [13]. By leveraging evidence‐based practices and interdisciplinary collaboration, these nurses ensure comprehensive and individualized care for each patient, focusing on stabilizing physiological functions and managing pain to promote recovery [14]. The implementation of effective pain management strategies, including both pharmacological and nonpharmacological approaches, is essential in ensuring patient comfort and facilitating the recovery process [15]. These interventions help prevent complications and promote early mobilization, leading to improved outcomes and shorter hospital stays [16].

Critical care environments, including GICUs and POCUs, rely on well‐organized teamwork to ensure optimal patient care. The complexity of these settings necessitates the collaboration of various healthcare professionals, each contributing their expertise to the management of critically ill or postoperative patients [17]. However, the absence of standardized protocols in some settings can lead to inefficiencies in patient admissions and care delivery [18]. A lack of clearly defined roles and responsibilities during the admission process can result in delays, inadequate preparations, and communication breakdowns, all of which negatively impact patient outcomes [19, 20]. The need for efficient processes in critical care settings has led to an increased interest in methodologies such as Lean management, which focuses on eliminating waste and improving the flow of care.

Lean methodology is based on defining value from the patient’s perspective, eliminating waste (non‐value‐adding activities), optimizing the work process, and continuous improvement (kaizen) [21–23]. Lean has been widely adopted in healthcare, where it aims to streamline clinical and administrative activities, reduce errors, shorten patient waiting times, and enhance overall service delivery [19, 20, 22, 24].

Past studies have shown a role of Lean methodology in intensive and postoperative care to standardize patient admission procedures, reduce the time of transfer, and define staff roles during high‐risk care transfers [22, 23, 25–27]. The applications are particularly useful when dealing with a time‐sensitive and high‐complex environment where coordination and efficiency are important in terms of patient outcomes. In the present study, however, Lean was used only as an assessment framework rather than an implemented intervention to compare existing practices in GICU and POCU settings.

For clarity, in this study, “Lean readiness” refers to the extent to which existing nursing workflows during patient admission already reflect Lean principles, that is, the proportion of value‐added (VA) versus non‐value‐added (NVA) activity, the presence or absence of standardized protocols, and the degree of waste (in time, effort, and cost) built into current practice. Assessing Lean readiness in this sense means diagnosing the current state of a process against Lean criteria; it does not involve introducing a Lean intervention or measuring the effect of one. This distinction is used consistently throughout the manuscript to differentiate the present assessment study from an implementation or improvement study. The concept is adapted from the notion of organizational readiness for change [28].

Research on the implementation of Lean in GICUs has reported improvements in efficiency and staff communication, including reduced turnaround times for patient admissions [8, 25]. Recent studies (published in the last 3–5 years) have reported that Lean frameworks were associated with shorter hospital stays, fewer safety incidents, and enhanced workflow satisfaction among clinical teams [29, 30]. Although the application of Lean in critical care is still an emerging field, initial studies suggest that it may improve outcomes by optimizing processes and minimizing waste [31–33]. However, the literature still lacks comprehensive comparative studies between traditional and Lean‐based patient admissions, especially in low‐resource healthcare settings such as Egypt. This gap is directly addressed in the current study.

The role of nurses in GICU and POCU settings extends beyond direct patient care to include a range of indirect tasks essential to the admission process, such as preparing equipment, ensuring the availability of necessary supplies, and facilitating communication among healthcare team members [19]. These indirect care activities often compete with direct patient care for nurses′ time, making efficient resource allocation crucial for optimizing admissions [34]. Recognizing the importance of both direct and indirect care enables healthcare institutions to develop targeted strategies for streamlining nursing workflows and improving patient outcomes [35].

In the context of GICU and POCU admissions, applying Lean principles may help streamline processes, reduce waste, and improve communication, potentially enhancing the efficiency and quality of care for critically ill and postoperative patients [24, 36]. This approach also aligns with the ethical principle of nonmaleficence by seeking to eliminate unnecessary burdens on patients and providers [25]. While further research is needed to evaluate the long‐term impact of Lean methodologies on admissions, early evidence suggests possible improvements in workflow and patient outcomes [37]. Integrating Lean into critical care settings may represent a step toward optimizing care delivery, fostering a culture of safety, and promoting collaboration within healthcare teams [38].

### 1.1. Research Significance and Problem of the Study

Efficient patient transfer and admission processes are critical to delivering safe, timely, and high‐quality care in high‐dependency areas such as GICU and POCU [39–41]. The vulnerability of patients during transitions of care, particularly following surgical procedures or upon GICU admission, heightens the risk of clinical deterioration, delays in critical interventions, and adverse outcomes [5, 17]. Traditional admission procedures in these units often suffer from inefficiencies, role ambiguity, communication breakdowns, and inconsistent adherence to clinical protocols, all of which contribute to delays and compromised patient care [9, 19].

This work acquires its importance due to the new use of Lean to evaluate and compare practices in patient admission at POCUs and GICUs in a low‐resource environment. In this regard, a low‐resource setting can be defined as healthcare settings with limited financial, technological, and human resources, which can include low staffing, low access to a high level of medical devices, and low budgets that could influence the adoption of standardized practice or quality improvement programs. A Lean assessment lens can reveal inefficiencies and non‐value‐adding activities and show where workflow design and team coordination could be improved [24, 32, 42]. Rather than revisiting Lean’s industrial origins, this study focuses on its growing relevance in healthcare, where it has been used to improve clinical processes and minimize waste [21–23, 42]. Although the literature on Lean in the critical care setting is increasingly global, the lack of empirical research aimed at the comparative evaluation of patient transfers into POCUs and GICUs based on structured Lean frameworks is evident in the Arab or developing healthcare context [43, 44].

Moreover, the significance of this study lies in its potential to contribute to the development of standardized, evidence‐based admission protocols tailored to the dynamic needs of postoperative and criticaly ill patients [38, 45]. By differentiating between direct and indirect nursing tasks and evaluating the overall flow and structure of current admission processes, the study emphasizes the importance of clear role delineation and interprofessional collaboration [34, 46]. This may enhance operational efficiency and support safer and more patient‐centered care. In addition, the findings of this study offer practical value for hospital administrators, nurse managers, and healthcare policymakers. The study provides data‐driven insights that can inform quality improvement initiatives, guide workforce training programs, and serve as a foundation for policy development aimed at enhancing care transitions [36, 37]. This is particularly important in the context of resource‐constrained hospitals, where optimizing the use of personnel and time is essential for sustainability and safety.

Another important dimension of this study is its relevance to nursing practice development. As frontline implementers of admission protocols, nurses are uniquely positioned to benefit from a Lean‐informed approach that clarifies responsibilities, reduces workload inefficiencies, and promotes a culture of accountability and continuous improvement [35]. Enhancing nurses’ involvement in structured and streamlined admissions can reduce stress, minimize errors, and improve patient–nurse interactions during critical phases of care. Ultimately, the study addresses a major gap in healthcare process optimization literature by providing comparative insights into how Lean principles might be applied to improve patient admissions in both POCU and GICU settings. Its implications extend beyond the immediate context of patient admissions to broader efforts in healthcare quality assurance, patient safety, staff satisfaction, and clinical outcome optimization [8, 38].

### 1.2. Aim of the Study

To assess the Lean readiness of patient admission processes in the GICU and POCU, focusing on the VA aspects of nursing practices, including time, cost, and effort during patient transfers.

Lean was used in this study as an assessment framework to describe current practice and Lean readiness. No Lean intervention was implemented; the study, therefore, cannot evaluate Lean‐driven improvement.

### 1.3. Objectives

1. To evaluate the Lean readiness of nursing practices during patient admissions in the GICU and POCU.
2. To assess the VA and NVA aspects of nursing activities during patient transfers, including time, cost, and effort.
3. To identify areas for potential improvement in patient admission processes based on Lean principles.

### 1.4. Research Questions

1. What is the Lean readiness of nursing practices during patient admissions to the GICU and POCU?
2. Which nursing activities are VA and NVA in the context of Lean principles, and how do they vary in terms of time, cost, and effort?
3. How can the findings inform strategies to optimize patient transfer processes and enhance efficiency using Lean methodology?

## 2. Materials and Method

### 2.1. Design and Participants

This study employed a descriptive comparative research design to systematically assess the Lean readiness of nursing practices during patient admissions in GICUs and POCUs. The aim of the study was to assess the Lean readiness of patient admission processes in the GICU and POCU, focusing on the VA aspects of nursing practices, including time, cost, and effort during patient transfers. This design was selected to enable detailed observation and quantification of naturally occurring nursing activities, focusing on VA and NVA practices in terms of time, cost, and effort. The study is reported in accordance with the STROBE statement for observational studies [47].

Participants included a convenience sample of 100 nurses, each actively involved in the patient admission process. The sample size was calculated using the Epi Info program [48], with parameters set at a 5% margin of error, a 99% confidence level, and an anticipated medium effect size of 0.5 [49], consistent with recommendations and practices in similar observational studies in clinical settings [50, 51]. Inclusion criteria required that participants be currently employed in the GICU or POCU and directly involved in patient admissions. Exclusion criteria included newly hired nurses with less than 3 months of experience, nurses on orientation or training, and those not routinely assigned to admission duties to ensure consistency in the practice level and familiarity with admission protocols.

#### 2.1.1. Independence of Observations, Sample Size, and Power

Each nurse was observed once, during one patient admission (100 nurses and 100 admissions); no nurse contributed more than one observation, and observations were treated as independent. Clustering of nurses within units and shifts was not modeled. The 47 GICU and 53 POCU nurses represent most of the nursing staff of the two units (see Study Settings). A sensitivity analysis for the achieved sample (*n* = 47 and 53; two‐sided *α* = 0.05) showed approximately 70% power to detect a medium standardized difference (*d* = 0.5) and 80% power for *d* ≥ 0.59 (allowing for the lower efficiency of the Mann–Whitney test); nonsignificant findings should therefore be interpreted cautiously.

### 2.2. Study Settings

This study was conducted in GICU and one POCU within the general surgical departments of a large university hospital in Egypt (AMUH). AMUH was selected due to its comprehensive facilities, high patient volume, and role as a major public teaching hospital in the region. The hospital is accredited by the General Authority for Healthcare Accreditation and Regulation (GAHAR) and manages thousands of surgical and critical care admissions annually. The GICU comprises 20 beds and employs approximately 50 nurses, primarily managing patients with life‐threatening conditions requiring advanced hemodynamic monitoring and ventilatory support. The POCU includes 15 beds and employs 53 nurses, focusing on receiving and stabilizing patients in the immediate postoperative period before transfer to surgical wards. Both units admit high‐risk patients, but differ in resources, policies, workflows, and staff qualifications. These functional distinctions influence the timing, complexity, and sequence of nursing care during patient admissions, which this study aimed to explore. By observing these units within a single large, publicly funded hospital serving a wide demographic of patients, the study provides insights into nursing practices and Lean readiness during patient transfers, reflecting real‐world clinical practice while maintaining participant anonymity.

### 2.3. Study Instruments

The study utilized a customized tool, “Lean Assessment of Receiving Patient to the Destination Unit Process—Observational Checklist,” developed by the researchers to evaluate Lean‐related nursing practices during patient admissions in the GICU and POCU. The tool was designed based on an extensive review of Lean healthcare literature, particularly studies addressing workflow efficiency, patient handover practices, and Lean applications in critical and perioperative care [40, 41, 52, 53].

The tool consists of two main sections:

 Part I collects demographic information from each nurse once at the beginning of their participation in the study. This includes gender, age, education, years of clinical and specialty experience, and prior training in Lean or critical care admissions.
 Part II documents and evaluates nursing practices during the patient admission process, covering eight specific categories of care activities: Preparation for receiving patient, use of a recognized assessment framework to assess patient status (e.g., ABCDE approach), measuring vital signs, wound/surgical site assessment, checking fluid status, managing pain, safety, and review of data present, notes, and charts.

Each activity was recorded with start and end times, enabling precise calculation of time consumed. Activities were further evaluated using a Lean‐based scoring system, categorizing them as VA or NVA based on established criteria and expert consensus. The tool also identifies eight types of Lean waste. Operationally, an activity was classified as VA when it directly contributed to assessing or caring for the patient (something the patient would be willing to receive) and as NVA when it consumed time, effort, or cost without benefiting the patient (for example, waiting, searching for equipment or information, duplicated documentation, or unnecessary movement). Time was recorded in minutes from the start to the end of each activity; effort and cost were recorded with the checklist rating scales and expressed as scores from 0 to 100.

### 2.4. Tool Development, Validity, and Reliability

The tool underwent content validity assessment by a panel of five subject matter experts in nursing practice and Lean healthcare systems. Their feedback informed iterative revisions to enhance clarity and comprehensiveness. Internal consistency was excellent (Cronbach’s alpha = 0.919; dimension‐specific coefficients 0.841–0.945) (see Supporting Table 8), indicating reliable measurement of intended constructs. Construct validity was supported by exploratory and confirmatory factor analyses (CFAs), which examined the factor structure of the checklist; the empirical structure only partly matched the eight a priori domains (see Results and Limitations) [54, 55]. Detailed item‐factor loadings and reliability measures are provided in Supporting File 1.

A new observational checklist was developed rather than adopting an existing Lean‐assessment tool because available instruments were designed for manufacturing or general hospital workflow contexts and did not adequately capture the specific, time‐critical activities performed during GICU and POCU admissions, such as structured ABCDE assessment and wound/surgical site checks. Items were generated through a structured process: an initial item pool was informed by the Lean‐healthcare and patient‐handover literature [40, 41, 52, 53] and the clinical experience of the research team and then reduced and refined by a five‐member expert panel, which reviewed the checklist for content validity. The resulting checklist comprised 74 items across eight dimensions: preparation for receiving the patient, assessment using a recognized framework (e.g., ABCDE), measurement of vital signs, wound/surgical site assessment, fluid‐status assessment, pain management, safety, and review of available data, notes, and charts.

Data were collected through direct observation by three raters. Before data collection, the observers received 1 week of training on the checklist items and scoring procedures. Nurse observers who were not part of the study sample assisted in scoring the observations after receiving the same training. A pilot study with 10 nurses (10% of the sample excluded from the analysis) was conducted to assess the checklist’s practical applicability, and no further refinements were needed after this phase.

### 2.5. Exploratory Factor Analysis (EFA) and CFA

An EFA using principal component extraction with Promax rotation and Kaiser normalization was used to examine the latent structure of the checklist [54]. A CFA specifying the eight a priori domains was then performed [55], and model fit was judged with CFI, IFI, and RMSEA [56]. Item loadings, communalities, item–total correlations, and correlations between domains are reported in the Results (psychometric properties of the observational checklist) and in Supporting Tables 1–8 and Supporting Figure 1.

### 2.6. Data Collection

Researchers underwent structured training on the checklist, time documentation, value assessment, and waste classification. Nonparticipant structured observation was used to assess nursing activities during 100 patient admissions in the GICU and POCU at a general University Hospital. Observations covered all shifts (morning, evening, and night) to ensure comprehensive coverage. Activities were classified as VA or NVA according to Lean principles [22]. Observers used unobtrusive techniques and, where possible, dual independent observations to enhance interobserver reliability. No patient demographic data were collected.

### 2.7. Ethical Considerations

This study received ethical approval from the Ethics and Research Committee of the Faculty of Nursing, Alexandria University, Egypt (IRB00013620 (30/9/2028), Approved Date:14‐10‐2020). Managers of participating nurses provided approval to facilitate observations, and verbal informed consent was obtained from all nurses prior to data collection. Participation was voluntary, and nurses were fully informed of the study purpose, procedures, and their right to withdraw at any time without repercussions; in the event of withdrawal, all data related to that nurse would be excluded from the study. Confidentiality and anonymity were maintained by assigning each nurse a unique code number for data recording, and all records were securely stored and accessible only to the research team.

### 2.8. Statistical Analysis

Data were analyzed using the IBM SPSS software package, Version 22.0 (Armonk, NY: IBM Corp). The analysis process began with assessing the distribution of variables using the Kolmogorov–Smirnov test to check for normality. Based on the results, nonparametric tests were chosen because the continuous variables did not meet the assumptions of normality, thus justifying the use of the Mann–Whitney *U* test instead of the independent samples *t*‐test. For categorical variables, comparisons between groups were conducted using the chi‐square test, with Fisher’s or Monte Carlo adjustments applied when necessary. The Mann–Whitney *U* test was used to compare nonnormally distributed quantitative variables between two groups. The significance of the results was determined at the 5% level. Regarding data completeness, the dataset was reviewed for missing values prior to analysis. Cases with missing data were minimal and were excluded listwise from the relevant analyses, as the proportion of missing data was not sufficient to warrant imputation. Effect sizes for the Mann–Whitney comparisons were expressed as r = Z/√N (0.1, 0.3, and 0.5 indicating small, medium, and large effects [49]). Ninety‐five percent confidence intervals for differences in means (Welch method) are reported descriptively to show the size of the differences; because the tests were rank based, the interval for a mean difference may include zero when the rank‐based test is significant in skewed data. Practical (managerial) significance was judged from the effect size and the size of the difference on the original scale rather than from *p* values alone. Because many comparisons were made, the *p* values for the eight individual activities were not adjusted; a Bonferroni‐adjusted threshold (0.05/8 ≈ 0.006) is given for reference, and the activity‐level findings are considered exploratory.

### 2.9. Methodological Rigor

This study used a descriptive comparative design to observe and compare nursing practices during patient admissions in the GICU and POCU without interfering with routine care. A convenience sample of 100 nurses was selected using Epi Info with a 5% margin of error, 99% confidence level, and a medium effect size of 0.5. Observations were conducted during various shifts namely, morning, evening, and night to ensure a comprehensive and balanced representation of nursing practices. Observations covered all three shifts although shift assignment was not randomized and most observed nurses worked morning shifts (55%; Table 1). The main tool used was the Lean Assessment of Receiving Patient to the Destination Unit Process Observational Checklist, which included demographic data, time‐stamped care activities, and classifications of VA and NVA activities based on Lean principles. The tool was validated by five experts and pilot tested with 10 nurses. It showed strong reliability (Cronbach’s alpha = 0.919) (Supporting Table 8) and validity through EFA and CFA. Structured nonparticipant observation was conducted by trained observers to reduce bias, and interobserver consistency was supported by dual observation and consensus (inter‐rater statistics were not computed; see Limitations). Data collection took place from October to December 2022 at the GICU and POCU of a general University Hospital, a GAHAR‐accredited center. Statistical analysis was performed using SPSS v22.0. The Kolmogorov–Smirnov test checked normality, and nonparametric tests (Mann–Whitney U, chi‐square, Fisher’s exact, and Monte Carlo) were used at a 5% significance level. Minimal missing data were excluded listwise. These steps supported the study’s validity, reliability, and ethical standards.

**TABLE 1:** Compression between the studied units according to nurse sociodemographic characteristics (*n* = 100).

Nurse characteristics	Total (*n* = 100)		Nurses in general intensive care unit (*n* = 47)		Nurses in postoperative care unit (*n* = 53)		*χ* ^2^	*p*
	No.	%	No.	%	No.	%		
*Nurses’ workplace*								
General intensive care unit	47	47	47	47	0	0		
Postoperative care unit	53	53	0	0	53	53	2.049	0.318

*Gender*								
Female	69	69.0	30	63.8	39	73.6	1.108	0.292
Male	31	31.0	17	36.2	14	26.4		

*Age (years)*								
Less than 20	26	26.0	15	31.9	11	20.8	2.755	0.431
Equal to 20 to less than 30	25	25.0	9	19.1	16	30.2		
Equal to 30 to less than 40	32	32.0	16	34.0	16	30.2		
Equal to or more than 40	17	17.0	7	14.9	10	18.9		

*Working shift*								
Morning	55	55.0	27	57.4	28	52.8	1.978	0.372
Evening	23	23.0	8	17.0	15	28.3		
Night	22	22.0	12	25.5	10	18.9		

*Level of education*								
Bachelor of nursing science	44	44.0	25	53.2	19	35.8	3.817	^MC^ *p* = 0.285
Diploma of technical health institute	29	29.0	13	27.7	16	30.2		
Diploma of secondary technical nursing	24	24.0	8	17.0	16	30.2		
Master of nursing science	3	3.0	1	2.1	2	3.8		

*Nursing years of experience*								
< 5	26	26.0	16	34.0	10	18.9	3.049	0.218
5 – 10	25	25.0	11	23.4	14	26.4		
> 10	49	49.0	20	42.6	29	54.7		

*Department specialty experience*								
< 5	26	26.0	13	27.7	13	24.5	5.169	0.075
5 – 10	43	43.0	15	31.9	28	52.8		
> 10	31	31.0	19	40.4	12	22.6		

*Educational experience*								
No	93	93.0	45	95.7	48	90.6	1.640	^MC^ *p* = 0.678
YES Lean management	2	2.0	1	2.1	1	1.9		
YES Receiving critical ill patient	5	5.0	1	2.1	4	7.5		

*Training experience about Lean management*								
No	93	93.0	45	93.6	49	92.5	0.052	^FE^ *p* = 1.000
Yes	7	7.0	2	6.4	4	7.5		

*Note:* *χ* ^2^: chi‐square test.

Abbreviations: FE, Fisher exact; MC, Monte Carlo.

## 3. Results

### 3.1. Characteristics of the Participants

Table 1 summarizes the sociodemographic and professional characteristics of the 100 nurses observed in this study. The sample included 47 nurses from the GICU and 53 from the POCU. The workforce was predominantly female in both units (GICU: 63.8%, POCU: 73.6%). The largest age group was 30–40 years (32%), and most nurses worked morning shifts (55%). Regarding education, 44% held bachelor’s degrees and 49% had more than 10 years of nursing experience. Nearly all nurses (93%) reported no prior training in Lean management or patient admission processes. The two units did not differ significantly in any demographic or professional characteristic (all *p* > 0.05; Table 1).

### 3.2. Psychometric Properties of the Observational Checklist

The EFA extracted eight components (Supporting Tables 1–4). Primary loadings ranged from 0.32 to 0.94 and communalities from 0.21 to 0.93. The empirically derived components did not map one‐to‐one onto the eight a priori domains: the 10 safety items (7.1–7.10) loaded together on one component (0.73–0.88), as did the bed and bed‐space preparation items (1.1.1–1.1.5; 0.81–0.91), whereas the vital‐sign items (3.4–3.10) and the wound/surgical site items (4.1–4.4) shared one component (0.52–0.94), and the ABCDE and fluid‐status items were distributed across several components. The CFA that specified the eight a priori domains (Supporting Figure 1) gave CFI = 1.000, IFI = 1.000, and RMSEA = 0.081, with a significant model test (1.751; *p* = 0.001). Fit indices of exactly 1.000, an RMSEA above the usual 0.06–0.08 range, and 100 observations for 74 items indicate that the CFA result must be interpreted with caution [56]. Corrected item–total correlations and correlations between domains are given in Supporting Tables 5–7. Internal consistency was high for the whole checklist (Cronbach’s alpha = 0.919; 74 items) and for the eight domains (alpha 0.841 to 0.945; Supporting Table 8).

### 3.3. Research Question 1: Lean Readiness of Admission Practices

Table 2 presents the mean scores for time, effort, and cost associated with key nursing activities during patient admissions, classified according to Lean assessment principles as VA or NVA. Activities such as using recognized assessment frameworks (e.g., ABCDE approach) and reviewing patient data were associated with the highest time and resource utilization, indicating their central role in the admission process. In contrast, preparatory activities for receiving patients consistently ranked lowest in time, effort, and cost, suggesting potential inefficiencies or opportunities for process improvement. The other indicators of Lean readiness were also limited: 93% of the nurses had no prior training in Lean management (Table 1), and the time per activity varied widely (standard deviation about 21 min around a mean of 24 min; Table 3), indicating inconsistent practice. Together, these results suggest that waste is measurable in all eight activities but that the processes and staff are only partly ready for Lean implementation.

**TABLE 2:** Comparison between overall mean score and the rank for observation of practices in general intensive care and postoperative care units in relation to their Lean assessment of receiving patient to the destination unit process (waste/non‐value added of care activities) (*n* = 100).

Themes of care and its activities	(Non‐value added)					
	Time		Effort		Cost	
	Mean ± SD	Rank	Mean ± SD	Rank	Mean ± SD	Rank
1. Preparation for receiving patient	67.92 ± 4.14	8	39.95 ± 5.22	8	7.99 ± 1.06	8
2. Use recognized assessment framework to assess patient status, e.g., ABCDE approach	96.78 ± 4.94	1	74.01 ± 6.74	7	59.10 ± 15.82	7
3. Measuring vital signs	93.75 ± 21.33	5	92.58 ± 23.92	4	92.50 ± 21.47	3
4. Wound/surgical site assessment	94.0 ± 20.45	4	91.50 ± 26.64	5	87.00 ± 27.40	6
5. Check fluid status	93.14 ± 23.37	7	93.86 ± 19.60	2	92.86 ± 21.54	2
6. Managing pain	94.67 ± 18.18	3	94.33 ± 17.77	1	87.33 ± 26.29	5
7. Safety	93.60 ± 21.81	6	93.30 ± 20.25	3	92.20 ± 20.28	4
8. Review data present, notes and charts	96.0 ± 13.63	2	90.75 ± 28.13	6	94.00 ± 20.45	1
Overall	89.77 ± 11.36		79.83 ± 12.65		69.09 ± 14.37	

*Note:* Time refers to the duration spent by nurses performing each activity, measured in minutes. Effort represents the physical or cognitive work required to complete the task. Cost indicates the resource utilization associated with the activity, including materials and staff time.

**TABLE 3:** Time consumed by nurses in total care practices for receiving patient to the destination unit.

Care practice activities of care and its activities	Time (start to end time)										
	Mean ± SD.	< 5		5–10		10–15		15–20		≥ 20	
		No.	%	No.	%	No.	%	No.	%	No.	%
1. Preparation for receiving patient	25.29 ± 22.33	16	16.0	7	7.0	10	10.0	9	9.0	58	58.0
2. Use recognized assessment framework to assess patient status	25.33 ± 22.34	21	21.0	6	6.0	12	12.0	0	0.0	61	61.0
3. Measuring vital signs	25.17 ± 22.43	16	16.0	10	10.0	7	7.0	12	12.0	55	55.0
4. Wound/surgical site assessment	25.17 ± 22.32	15	15.0	11	11.0	7	7.0	12	12.0	55	55.0
5. Check fluid status	25.53 ± 22.16	14	14.0	12	12.0	0	0.0	16	16.0	58	58.0
6. Managing pain	25.14 ± 22.37	16	16.0	10	10.0	7	7.0	12	12.0	55	55.0
7. Safety	25.09 ± 22.39	14	14.0	12	12.0	5	5.0	14	14.0	55	55.0
8. Review data present, notes and charts	25.03 ± 22.46	16	16.0	10	10.0	7	7.0	12	12.0	55	55.0
Overall	24.34 ± 21.17	15	15.0	12	12.0	6	6.0	12	12.0	55	55.0

*Note:* Time in minutes.

### 3.4. Research Question 2: VA and NVA Activities and Differences Between Units

Table 4 compares the time, effort, and cost scores between the units. Overall, the GICU scores were significantly higher than the POCU scores for time (90.9 ± 8.6 vs. 88.8 ± 13.4; *p* < 0.001; *r* = 0.49), effort (82.3 ± 7.1 vs. 77.7 ± 15.8; *p* = 0.006; *r* = 0.27), and cost (70.4 ± 13.2 vs. 68.0 ± 15.4; *p* = 0.001; *r* = 0.33), but the absolute differences were small (2.1, 4.6, and 2.4 points). At the activity level, the cost scores of wound/surgical site assessment (95.7 ± 16.8 vs. 79.3 ± 32.4; *p* = 0.003; *r* = 0.30) and managing pain (94.3 ± 18.8 vs. 81.1 ± 30.3; *p* = 0.011; *r* = 0.25) were higher in the GICU, and the effort score for preparation for receiving the patient differed significantly (*p* = 0.016; *r* = 0.24) although the means were almost identical (40.3 vs. 39.6). The time score for the use of a recognized assessment framework was significantly higher in the POCU (98.8 ± 3.4 vs. 94.5 ± 5.5; *p* < 0.001; *r* = 0.37). After Bonferroni adjustment for the eight activities (*α* ≈ 0.006), only this time difference and the wound/surgical site cost difference remained significant; no significant differences were found for safety or the remaining activities.

**TABLE 4:** Comparison of the mean values and ranks of the non‐value added to the practices during patients’ admission process to the general intensive care and postoperative care units.

Care practice items/activities	(Non‐value‐added categories)												*U* (p_1_)	*U* (p_2_)	*U* (p_3_)
	Nurses in general intensive care unit (*n* = 47)						Nurses in postoperative care unit (*n* = 53)								
	Time		Effort		Cost		Time		Effort		Cost				
	Mean ± SD	Rank	Mean ± SD	Rank	Mean ± SD	Rank	Mean ± SD	Rank	Mean ± SD	Rank	Mean ± SD	Rank			
1. Preparation for receiving patient	69.18 ± 4.70	8	40.31 ± 6.04	8	8.04 ± 1.14	8	66.81 ± 3.23	8	39.64 ± 4.41	8	7.94 ± 1.00	8	983.00 (0.058)	912.00^∗^ (0.016^∗^)	1083.50 (0.244)
2. Use recognized assessment framework to assess patient status,	94.50 ± 5.45	7	75.01 ± 7.24	7	56.98 ± 16.80	7	98.81 ± 3.36	1	73.13 ± 6.20	7	60.98 ± 14.79	7	706.50^∗^ (< 0.001^∗^)	1027.0 (0.088)	1049.50 (0.123)
3. Measuring vital signs	96.45 ± 17.00	5	97.87 ± 8.24	1	93.97 ± 20.02	5	91.35 ± 24.46	5	87.89 ± 31.33	4	91.19 ± 22.79	3	1163.50 (0.229)	1150.0 (0.205)	1194.0 (0.512)
4. Wound/surgical site assessment	96.81 ± 15.30	3	96.81 ± 12.35	4	95.74 ± 16.75	2	91.51 ± 23.99	4	86.79 ± 34.18	5	79.25 ± 32.42	6	1157.50 (0.196)	1150.0 (0.205)	947.0^∗^ (0.003^∗^)
5. Check fluid status	96.35 ± 17.49	6	97.26 ± 10.59	3	93.92 ± 20.15	6	90.30 ± 27.42	7	90.84 ± 24.75	2	91.91 ± 22.85	1	1159.50 (0.207)	1150.0 (0.205)	1210.50 (0.642)
6. Managing pain	97.16 ± 13.60	2	97.87 ± 8.24	1	94.33 ± 18.80	4	92.45 ± 21.33	3	91.19 ± 22.80	1	81.13 ± 30.32	5	1155.5 (0.186)	1150.0 (0.205)	993.0^∗^ (0.011^∗^)
7. Safety	96.60 ± 16.32	4	96.17 ± 14.83	5	94.89 ± 16.92	3	90.94 ± 25.59	6	90.75 ± 23.93	3	89.81 ± 22.74	4	1157.5 (0.196)	1150.0 (0.205)	1140.0 (0.211)
8. Review data present, notes and charts	97.87 ± 10.20	1	95.21 ± 18.53	6	96.81 ± 15.30	1	94.34 ± 15.99	2	86.79 ± 34.18	5	91.51 ± 23.99	2	1157.5 (0.196)	1150.0 (0.205)	1157.5 (0.196)
Overall	90.88 ± 8.60		82.29 ± 7.07		70.38 ± 13.19		88.78 ± 13.35		77.65 ± 15.82		67.95 ± 15.38		531.0^∗^ (< 0.001^∗^)	866.0^∗^ (0.006^∗^)	784.0^∗^ (0.001^∗^)

*Note:* U: Mann–Whitney test, p_1_: *p* value for comparing between the studied units (Time), p_2_: *p* value for comparing between the studied units (Effort), p_2_: *p* value for comparing between the studied units (cost).

^∗^Statistically significant at *p* ≤ 0.05.

Table 5 contrasts the VA and NVA scores of the two units. The use of a recognized assessment framework had a significantly higher VA score in the GICU than that in the POCU (89.6 ± 7.8 vs. 83.8 ± 2.4; mean difference 5.8, 95% CI 3.4–8.2; *p* < 0.001; *r* = 0.41, a medium‐to‐large effect), and the overall VA score was also higher in the GICU (97.0 ± 2.4 vs. 95.9 ± 2.1; mean difference 1.15, 95% CI 0.2–2.1; *p* = 0.037; *r* = 0.21, a small effect). Overall NVA scores were similar (36.4 ± 1.5 vs. 36.8 ± 1.5), and no other activity differed significantly in VA or NVA scores (all *p* > 0.05). Thus, the clearest difference between the units concerned the structured assessment, whereas the overall difference in VA was about one point on a 100‐point scale and is of limited practical size; NVA was present in both units, indicating areas for potential workflow optimization.

**TABLE 5:** Comparison between mean scores of the value and non‐value added to the nurses practices during patients’ admission process to the general intensive care and postoperative care units.

Care practice items/activities	Total (*n* = 100)		General intensive care unit (*n* = 47)		Postoperative care unit (*n* = 53)		*U*	*p*
	Non value added	Value‐ added	Non‐value added	Value added	Non‐value added	Value added		
	Mean ± SD.	Mean ± SD.	Mean ± SD.	Mean ± SD.	Mean ± SD.	Mean ± SD.		
1. Preparation for receiving patient	37.60 ± 0.21	97.24 ± 3.92	37.50 ± 0.0	96.25 ± 4.16	37.64 ± 0.24	98.19 ± 3.48	1112.50	0.295
2. Use recognized assessment framework to assess patient status	47.29 ± 0.16	86.62 ± 6.38	47.37 ± 0.00	89.58 ± 7.78	47.26 ± 0.18	83.78 ± 2.42	650.00^∗^	< 0.001^∗^
3. Measuring vital signs	30.80 ± 5.61	98.80 ± 5.80	30.56 ± 4.81	99.62 ± 2.51	30.90 ± 6.28	98.01 ± 7.69	1116.50	0.140
4. Wound/surgical site assessment	20.00 ± 10.54	98.33 ± 11.12	16.67 ± 14.43	99.43 ± 3.77	21.43 ± 9.45	97.28 ± 15.12	1138.50	0.206
5. Check fluid status	42.90 ± 30.09	98.42 ± 10.56	52.38 ± 41.24	98.38 ± 10.77	38.84 ± 26.97	98.46 ± 10.47	1163.50	0.276
6. Managing pain	46.63 ± 28.13	98.51 ± 9.91	55.56 ± 38.49	98.48 ± 10.05	42.81 ± 25.22	98.54 ± 9.88	1157.50	0.243
7. Safety	52.00 ± 25.30	98.67 ± 8.89	70.0 ± 34.64	98.60 ± 9.15	40.0 ± 0.0	98.72 ± 8.75	1160.50	0.259
8. Review data present, notes and charts	47.50 ± 36.23	97.50 ± 13.54	50.00 ± 43.30	98.30 ± 11.31	46.43 ± 36.60	96.74 ± 15.46	1160.50	0.259
Overall	36.67 ± 1.44	96.45 ± 2.33	36.44 ± 1.54	97.04 ± 2.44	36.76 ± 1.51	95.89 ± 2.10	953.50^∗^	0.037^∗^

*Note: U*: Mann–Whitney test, *p*: *p* value for comparing between the studied units.

^∗^Statistically significant at *p* ≤ 0.05.

Table 3 shows that the mean time per activity was about 25 min for each of the eight activities (overall 24.3 ± 21.2 min). The large standard deviations, and the fact that 55% of the observations lasted 20 min or longer, indicate wide variation in the time taken; no single activity took clearly longer than the others.

Table 6 compares the time in minutes between the units. For every activity the mean time was slightly shorter in the GICU than in the POCU (overall 22.9 ± 18.2 vs. 25.6 ± 23.6 min; mean difference −2.8 min, 95% CI −11.1–5.5), and the distribution of time categories did not differ significantly between the units for any activity (all *p* ≥ 0.22; overall *p* = 0.528). In terms of minutes, therefore, the admission practices of the two units were similar.

**TABLE 6:** Compression between time consumed by nurses in general intensive care and post‐operative care units in relation to their Lean assessment of receiving patient to the destination unit process.

Care practice activities of care and its activities	Time (start to end time)																						*χ* ^2^	^MC^ *p*
	General intensive care unit (*n* = 47)											Postoperative care unit (*n* = 53)												
	Mean ± SD.	< 5		5–10		10–15		15–20		≥ 20		Mean ± SD.	< 5		5–10		10–15		15–20		≥ 20			
		No.	%	No.	%	No.	%	No.	%	No.	%		No.	%	No.	%	No.	%	No.	%	No.	%		
1. Preparation for receiving patient	24.87 ± 21.11	9	19.1	4	8.5	3	6.4	5	10.6	26	55.3	25.66 ± 23.55	7	13.2	3	5.7	7	13.2	4	7.5	32	60.4	2.424	0.692
2. Use recognized assessment framework to assess patient status	24.87 ± 21.09	12	25.5	3	6.4	3	6.4	0	0.0	29	61.7	25.74 ± 23.58	9	17.0	3	5.7	9	17.0	0	0.0	32	60.4	3.231	0.355
3. Measuring vital signs	24.62 ± 21.27	9	19.1	6	12.8	1	2.1	6	12.8	25	53.2	25.66 ± 23.61	7	13.2	4	7.5	6	11.3	6	11.3	30	56.6	4.247	0.377
4. Wound/surgical site assessment	24.68 ± 21.11	9	19.1	6	12.8	1	2.1	6	12.8	25	53.2	25.60 ± 23.52	6	11.3	5	9.4	6	11.3	6	11.3	30	56.6	4.292	0.372
5. Check fluid status	25.06 ± 20.95	8	17.0	7	14.9	0	0.0	5	10.6	27	57.4	25.94 ± 23.37	6	11.3	5	9.4	0	0.0	11	20.8	31	58.5	2.795	0.424
6. Managing pain	24.60 ± 21.18	9	19.1	6	12.8	1	2.1	6	12.8	25	53.2	25.62 ± 23.57	7	13.2	4	7.5	6	11.3	6	11.3	30	56.6	4.247	0.377
7. Safety	24.60 ± 21.22	8	17.0	7	14.9	0	0.0	7	14.9	25	53.2	25.53 ± 23.57	6	11.3	5	9.4	5	9.4	7	13.2	30	56.6	5.668	0.220
8. Review data present, notes and charts	24.51 ± 21.30	9	19.1	6	12.8	1	2.1	6	12.8	25	53.2	25.49 ± 23.64	7	13.2	4	7.5	6	11.3	6	11.3	30	56.6	4.247	0.377
Overall	22.87 ± 18.19	9	19.1	6	12.8	1	2.1	6	12.8	25	53.2	25.64 ± 23.59	6	11.3	6	11.3	5	9.4	6	11.3	30	56.6	3.281	0.528

*Note:* *χ* ^2^: chi‐square test.

Abbreviations: FE, Fisher exact; MC, Monte Carlo.

In summary, the GICU showed a higher VA contribution of structured assessment and slightly higher overall time, effort, and cost scores, whereas the time in minutes was similar in the two units. Most differences were small in size (Table 7), and NVA activity was present in both units.

### 3.5. Research Question 3: Implications for Optimizing Patient Transfer Processes

Figure 1 presents the workflow proposed by the research team in response to this question. It is a conceptual synthesis: the left‐side column summarizes the types of waste (time, effort, cost, or mixed) attributed to the eight activities, and the right‐side column pairs each activity with Lean tools and practices described in the literature. The proposed workflow was not derived statistically from the observational data, was not implemented, and was not tested, and no improvement estimates were measured in this study. The suggested pilot sequence (POCU first and then GICU) is a recommendation for future work and differs from the instrument‐testing pilot described in the Methods.

**FIGURE 1:**
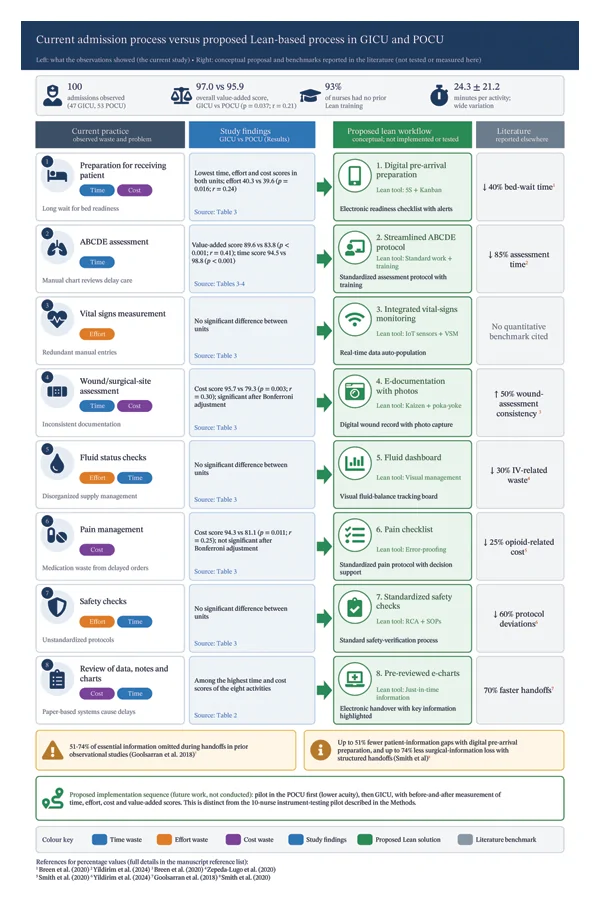
Current admission process versus proposed Lean‐based process in GICU and POCU. Left: practice observed in this study with the corresponding findings (GICU vs. POCU mean scores; *r* = effect size). Right: conceptual Lean workflow proposed by the research team, with benchmarks reported in the cited literature. None of the proposed steps was implemented or tested in this study, and the percentages were not measured here.

## 4. Discussion

This study assessed the Lean readiness of patient admission practices in a GICU and a POCU of one university hospital. Lean was not implemented, so the findings describe current practice and cannot show that Lean improves admissions. The principal findings were that (1) the GICU showed a higher VA contribution of structured assessment, (2) overall time, effort, and cost scores were slightly higher in the GICU, (3) the time in minutes was similar in the two units and highly variable, and (4) most nurses had no Lean training. Taken together, these findings are consistent with partial Lean readiness, as explained in the following.

Lean readiness can be judged from the three indicators defined in the Introduction. First, waste was measurable in every activity in both units, so the first Lean step, making waste visible, is feasible with the present checklist [24, 36]. Second, standardization was uneven: the structured ABCDE assessment in the GICU was associated with a higher VA score and a lower time score than that in the POCU (94.5 vs. 98.8; Table 4), whereas the time per activity varied widely in both units (standard deviation about 21 min around a mean of 24 min), suggesting that work is not yet performed to a common standard; stable, standardized work is a prerequisite for Lean improvement [22, 23]. Third, 93% of the nurses had no Lean education or training, so the workforce is not yet prepared to identify and remove waste itself. Preparation for receiving the patient had the lowest time, effort, and cost scores in both units; this could mean that preparation is efficient or that it is under‐resourced, and the checklist cannot distinguish between the two, so process mapping and staff interviews are needed before conclusions are drawn.

The differences between the units are plausibly related to case mix and unit organization. GICU patients are typically unstable and need a full, structured primary survey and more intensive monitoring, which may explain the higher VA score for assessment and the higher cost scores of wound/surgical site assessment and pain management, which involve more devices, dressings, and medication in complex patients [1, 35]. In the POCU, the higher time score together with the lower VA score for the assessment framework may reflect a less structured use of the ABCDE approach because assessment and handover in postoperative areas often follow local habits rather than a checklist [19, 40]. Patient acuity, staffing ratios, and unit policies were not recorded, so these explanations remain hypotheses, and the differences may be confounded by case mix or by nurse‐to‐patient ratios.

Most between‐unit differences were small. The overall mean differences were 2–5 points on a 0–100 scale, and the overall VA difference was about one point although the rank‐based effect sizes were small to medium (*r* = 0.21–0.49), and the differences in minutes were not significant (Table 6). Differences of this size are unlikely, by themselves, to justify different redesigns for the two units. The main managerial signal is the finding common to both units of wide variability in time per activity and of NVA components in every activity, which supports unit‐specific standard work rather than a comparison between units. The study also had limited power to detect small differences, so nonsignificant findings do not prove equivalence.

Several organizational and staffing implications follow for nurse managers. First, a structured, unit‐specific admission standard, for example, a nurse‐led ABCDE checklist in the POCU, could reduce variation in practice [40, 41]. Second, because 93% of the nurses had no Lean training, brief Lean and quality‐improvement education should be added to orientation and continuing education so that frontline nurses can identify waste. Third, admissions compete with other direct and indirect care for nurses’ time [34, 35], so staffing for admissions could be planned around expected arrivals, for example, by assigning a second or floating nurse and by standardizing equipment readiness. Fourth, standardized documentation and handoff tools such as SBAR or ISBAR may reduce the time spent on chart review and duplicated entries [40, 41, 53]; in our data, reviewing data, notes, and charts had one of the highest time and cost scores, but the data cannot show how much time such tools would save.

Several barriers may affect Lean implementation in this and similar low‐resource hospitals. Limited funding and information technology may prevent digital prearrival coordination or electronic documentation; high workload and staff shortages leave little time for improvement work; staff with no Lean exposure may see Lean as extra work; and admissions depend on the operating room, anesthesia, and transport teams, so improvement cannot be achieved by nursing alone [18, 19]. Leadership support, frontline engagement, and a culture of continuous improvement (kaizen) are generally needed to sustain Lean changes [36, 37]. Low‐cost steps such as standard work, visual management, and checklists may be more feasible than technology‐dependent solutions; Farghaly and Ahmed [42] describe a Lean application in an Egyptian intensive care setting.

Figure 1 was developed by the research team as a conceptual proposal. It was informed by the activities in which the observations showed time, effort, or cost waste and by Lean tools described in the literature, but it did not emerge from a formal analysis of the observational data, and it has not been implemented or tested. Its value is as a starting point for local improvement teams, who should adapt it to local resources and test it, for example, in a small pilot in one unit (the POCU first, because of its lower acuity, and then the GICU) with before‐and‐after measurement of the time, effort, cost, and VA scores using the present checklist. Improvements reported in other settings cannot be assumed to apply in Egyptian hospitals.

## 5. Conclusion

This assessment showed that nursing activities during admissions to the GICU and POCU differed modestly in VA contribution and in time, effort, and cost scores, while the time in minutes was similar. The GICU showed a higher VA contribution of structured assessment, at slightly higher resource scores. No Lean intervention was implemented, so the study does not show that Lean improves admissions. In relation to the aim, the findings indicate partial Lean readiness: waste is measurable in both units, but practice is variable, standardization is uneven, and most nurses have no Lean training. Unit‐specific standard work and staff education are reasonable first steps, and their effect on efficiency and patient safety should be tested in intervention studies.

## 6. Future Research

Future research should test the workflow proposed in Figure 1 in a pilot or stepped quality‐improvement study with before‐and‐after measurement of time, effort, cost, and VA scores and should evaluate its effect on patient outcomes and staff workload. Multicenter studies with larger samples would allow adjustment for patient acuity, staffing, and the clustering of nurses within units and shifts and would permit a more robust CFA and full reporting of the inter‐rater reliability of the checklist. Qualitative methods such as interviews or focus groups would clarify staff perceptions and barriers to Lean implementation. Finally, research on interprofessional coordination between the operating room, POCU, and GICU, and on digital tools and decision support [2], could help to streamline assessments and care transitions.

## 7. Implications to Practice

This research paper demonstrates that measuring value and nonvalue activities in the process of patient admission can reveal the existing differences in workflows and the existence of certain inefficiencies within POCU and GICUs. The results point out that formal assessment frameworks and standardized protocols were associated with higher VA care, specifically in the GICU, although they might also involve higher resource expenditure [35, 45].

Through the systematic review of the existing admission processes, healthcare teams can identify aspects in which the workflow can be improved and the number of nonvalue‐adding tasks decreased. Studies of Lean healthcare interventions suggest that protocol improvement and waste reduction can improve efficiency and resource utilization although the quality of the evidence varies [24, 36, 44].

Although Lean interventions and training were not applied in this study, the comparative assessment approach provides a background for future quality improvement activities. Hospitals can look into applying similar observational assessments to drive specific process changes and to influence the formulation of evidence‐based strategies on safer and more efficient patient admissions [36, 44].

## 8. Implications to Nursing Management

The findings offer nurse leaders a practical starting point for moving from routine supervision toward Lean leadership. Because the units differ in patient acuity and resources, a single standardized protocol for both is unlikely to fit; leaders should rather support unit‐specific standard work that matches the resource needs of the GICU and the POCU.

Because 93% of the nurses had no Lean training, nurse managers should include quality‐improvement and Lean content in orientation and continuing education so that nurses can detect and report wasteful activities in their own workflows.

Management support may also be needed for standard digital handoff tools and for better inventory and equipment management, which may reduce NVA search time and redundant information transfer between teams. At the system level, staffing models and resource allocation could be aligned with unit‐level Lean readiness rather than applied uniformly. A frontline‐driven kaizen culture may contribute to more efficient use of resources and to staff satisfaction, but these benefits were not measured in this study.

## 9. Limitations

Several limitations should be considered when interpreting these findings. First, this was an observational assessment in a single general university hospital with a convenience sample of 100 nurses, so the findings may not be generalizable to other hospitals, countries, or private or lower‐acuity settings (limited external validity). Second, no Lean intervention or test of the processes was carried out; Lean served only as an assessment framework, and no conclusion about improvement attributable to Lean can be drawn. Third, each nurse was observed once, during one admission, and nurses knew they were being observed, so a Hawthorne effect cannot be excluded [57]. Shifts were not randomized, and the clustering of nurses within units and shifts was not modeled. Fourth, patient acuity, staffing ratios, and unit policies were not recorded, so differences between the units may reflect case mix or organization rather than nursing practice.

Fifth, the checklist is a new instrument, and its psychometric properties are preliminary. The sample (*n* = 100) was small for a factor analysis of 74 items, the empirical factors did not match the a priori domains, and the CFA fit indices (CFI and IFI = 1.000) suggest overfitting. In addition, although the five‐member expert panel reviewed the checklist for content validity, item‐level and scale‐level content validity indices were not calculated [58]. Inter‐rater reliability statistics (e.g., Fleiss’ kappa or intraclass correlation coefficients) were also not computed for the three raters or the assisting nurse observers. Observer consistency was, therefore, supported by a 1‐week training program and a pilot phase with 10 nurses rather than by statistical agreement estimates, so observer variability and observer bias cannot be excluded. The checklist should be validated in a larger independent sample that reports content validity indices and inter‐rater reliability.

Sixth, the study was powered for a medium effect and many comparisons were made without adjustment, so activity‐level findings are exploratory and small differences may have gone undetected. Finally, only quantitative data were collected, which prevents exploration of the staff perceptions and experiences that influence workflow optimization. Situational conditions such as hospital policies, administrative support, and staffing may also have influenced the observed practices.

## Funding

There was no funding supporting the current study.

## Ethics Statement

This study received ethical approval from the Ethics and Research Committee of the Faculty of Nursing, Alexandria University, Egypt (IRB00013620 (30/9/2028), Approved Date: 14‐10‐2020).

## Consent

Verbal informed consent was obtained from all participating nurses, and the unit managers approved the observations to facilitate the research process. To uphold the confidentiality and anonymity of the study subjects, rigorous measures were taken to protect their identities and ensure the secure handling of data. The privacy of participants’ responses was strictly maintained throughout the study. Prior to inclusion, participants were thoroughly informed about their right to voluntary participation and their ability to withdraw from the study at any time without any repercussions. These ethical practices were diligently followed to ensure the study’s integrity and the protection of participants’ rights.

## Conflicts of Interest

The authors declare no conflicts of interest.

## Supporting Information

Additional supporting information can be found online in the Supporting Information section.

## Supporting information


**Supporting Information** Supporting Table 1: Exploratory factor analysis and factor loadings EFA. Supporting Table 2: Exploratory factor analysis and factor loadings EFA. Supporting Table 3: Exploratory factor analysis and factor loadings EFA. Supporting Table 4: Exploratory factor analysis and factor loadings EFA. Supporting Figure 1: CFA by structure equation modeling *n* = 100. Supporting Table 5: Corrected item–total correlations. Supporting Table 6: Corrected item–total correlations. Supporting Table 7: Correlations between dimensions. Supporting Table 8: Cronbach’s alpha.

## Data Availability

The data that support the findings of this study are available on request from the corresponding author. The data are not publicly available due to privacy or ethical restrictions.
