# Supplementary material for: Revamping Patient Transfers: A Comparative Assessment of Lean Readiness in Intensive Care and Postoperative Unit Admissions
Source: J Nurs Manag. 2026 Nov 13;2026:1152328. doi: 10.1155/jonm/1152328 (PMC13768865; doi:10.1155/jonm/1152328)
Supplement: Supplementary file 1 [file JONM-2026-1152328-s001.docx]

**Tool development Lean assessment of receiving patient to the destination unit process**

**Supplementary Table (1): Exploratory factor analysis and factor loadings (EFA)**

|  |  | **Principal Component Analysis.** | | | | | | | |
| --- | --- | --- | --- | --- | --- | --- | --- | --- | --- |
| **Item number** | **Section** | **Factor 1** | **Factor 2** | **Factor 3** | **Factor 4** | **Factor 5** | **Factor 6** | **Factor 7** | **Factor 8** |
| **1-Preparation for receiving patient** | | | | | | | | | |
| **1.1 Bed and the bed space preparation:** | | | | | | | | | |
| item1 | 1.1.1 | 0.377 | 0.302 |  | 0.693 |  |  |  |  |
| item2 | 1.1.2 | 0.374 |  |  | 0.661 |  |  |  |  |
| item3 | 1.1.3 | 0.309 | 0.327 |  | 0.534 |  | 0.340 |  |  |
| item4 | 1.1.4 | 0.454 | 0.306 |  | 0.464 |  |  |  |  |
| item5 | 1.1.5 | 0.487 |  |  | 0.512 |  |  | 0.330 |  |
| **1.2 Emergency equipment / machines preparation:** | | | | | | | | | |
| item6 | 1.2.1 | 0.477 | 0.493 |  | 0.368 |  |  |  |  |
| item7 | 1.2.2 | 0.518 | 0.311 |  | 0.462 |  |  |  |  |
| item8 | 1.2.3 | 0.437 | 0.441 |  | 0.544 |  |  |  |  |
| item9 | 1.2.4 | 0.483 | 0.319 |  | 0.432 |  |  |  |  |
| item10 | 1.2.5 | 0.374 |  |  |  |  | 0.425 | 0.322 |  |
| item11 | 1.2.5.1 | 0.558 | 0.310 |  |  |  | 0.496 |  |  |
| item12 | 1.2.5.2 | 0.440 |  |  | 0.326 |  |  | 0.381 |  |
| item13 | 1.2.6 | 0.396 |  |  |  |  |  |  |  |
| item14 | 1.2.6.1 |  |  |  |  | 0.305 |  |  |  |
| item15 | 1.2.7 | 0.523 |  |  |  | 0.422 |  |  |  |
| item16 | **1.3** | 0.395 |  |  |  |  |  | 0.332 | -0.421 |
| item17 | **1.4** | 0.422 |  | 0.367 |  |  |  | 0.499 |  |
| **2-Use recognized assessment framework to assess patient status, e.g. ABCDE approach** | | | | | | | | | |
| ***2.1 Airway:*** | | | | | | | | | |
| item18 | 2.1.1 | 0.430 |  | 0.416 |  |  |  | 0.520 |  |
| item19 | 2.1.2 |  | 0.383 | 0.372 |  |  |  | 0.358 |  |
| item20 | 2.1.3 |  | 0.424 | 0.351 |  |  |  | 0.339 |  |
| item21 | 2.1.4 | 0.413 | 0.345 | 0.401 |  |  |  |  | 0.438 |
| ***2.2 Breathing*** | | | | | | | | | |
| item22 | 2.2.1 | 0.390 | 0.301 | 0.427 |  |  |  |  | 0.436 |
| item23 | 2.2.2 | 0.487 | 0.416 | 0.434 |  |  |  |  |  |
| ***2.3 Circulation : Assess the patient’s circulation via*** | | | | | | | | | |
| item24 | 2.3.1 |  | 0.361 | 0.594 |  |  | 0.447 |  |  |
| item25 | 2.3.2 |  | 0.359 | 0.553 |  |  | 0.455 |  |  |
| item26 | 2.3.3 | 0.508 | 0.424 | 0.403 |  |  |  |  |  |
| item27 | 2.3.4 | 0.348 |  |  |  | 0.354 | -0.406 |  |  |
| item28 | 2.3.5 | 0.445 |  |  |  | 0.448 | -0.387 |  |  |
| item29 | 2.3.6 | 0.337 | 0.314 |  |  | 0.316 |  | -0.311 |  |
| ***2.4 Disability*** | | | | | | | | | |
| item30 | 2.4.1 | 0.402 |  |  |  |  |  |  |  |
| item31 | 2.4.2 | 0.304 |  |  |  |  |  |  |  |
| item32 | 2.4.3 | 0.580 |  |  |  |  |  |  |  |
| ***2.5 Exposure / environmental control*** | | | | | | | | | |
| item33 | 2.5.1 | 0.446 | 0.336 |  |  |  |  | -0.400 |  |
| item34 | 2.5.2 | 0.640 | 0.419 | 0.381 |  |  |  |  |  |
| item35 | ***2.6*** | 0.575 | 0.423 | 0.431 |  |  |  |  |  |
| item36 | ***2.7*** | 0.596 | 0.428 | 0.427 |  |  |  |  |  |
| **3-Measuring vital signs** | | | | | | | | | |
| item37 | 3.1 | 0.691 | 0.395 | 0.399 |  |  |  |  |  |
| item38 | 3.2 | 0.664 | 0.428 | 0.402 |  |  |  |  |  |
| item39 | 3.3 | 0.648 | 0.439 | 0.394 |  |  |  |  |  |
| item40 | 3.4 | 0.509 | 0.598 | 0.398 |  |  |  |  |  |
| item41 | 3.5 | 0.393 | 0.614 | 0.362 |  |  |  |  |  |
| item42 | 3.6 | 0.447 | 0.424 | 0.499 | -0.412 |  | 0.306 |  |  |
| item43 | 3.7 | 0.469 | 0.402 | 0.475 | -0.396 |  | 0.307 |  |  |
| item44 | 3.8 | 0.469 | 0.662 | 0.389 |  |  |  |  |  |
| item45 | 3.9 | 0.419 | 0.656 | 0.343 |  |  |  |  |  |
| item46 | 3.10 | 0.466 | 0.663 |  |  |  |  |  |  |

**Supplementary Table (2): Exploratory factor analysis and factor loadings (EFA)**

|  |  | **Principal Component Analysis.** | | | | | | | |
| --- | --- | --- | --- | --- | --- | --- | --- | --- | --- |
| **Item number** | **Section** | **Factor 1** | **Factor 2** | **Factor 3** | **Factor 4** | **Factor 5** | **Factor 6** | **Factor 7** | **Factor 8** |
| **4-Wound/surgical site assessment** | | | | | | | | | |
| item47 | 4.1 | 0.481 | 0.716 |  |  |  |  |  |  |
| item48 | 4.2 | 0.495 | 0.632 |  |  |  |  |  |  |
| item49 | 4.3 | 0.472 | 0.391 |  |  |  |  |  | 0.360 |
| item50 | 4.4 | 0.645 |  |  |  |  |  |  | 0.434 |
| **5-Check fluid status** | | | | | | | | | |
| item51 | 5.1 | 0.653 | 0.362 |  |  |  |  |  | 0.350 |
| item52 | 5.2 | 0.596 | 0.426 |  | 0.330 |  |  |  | 0.397 |
| item53 | 5.3 | 0.613 | 0.392 |  | 0.372 |  |  |  | 0.302 |
| item54 | 5.4 | 0.533 | 0.601 |  |  |  |  |  |  |
| item55 | 5.5 | 0.450 |  |  |  | 0.722 |  |  |  |
| item56 | 5.6 | 0.387 | 0.305 |  |  | 0.641 |  |  |  |
| item57 | 5.7 | 0.428 |  |  |  | 0.726 |  |  |  |
| **6-Managing pain** | | | | | | | | | |
| item58 | 6.1 | 0.580 |  | 0.655 |  |  |  |  |  |
| item59 | 6.2 | 0.637 |  | 0.518 |  |  |  |  |  |
| item60 | 6.3 | 0.565 |  | 0.659 |  |  |  |  |  |
| **7-Safety** | | | | | | | | | |
| item61 | 7.1 | 0.593 | 0.500 |  |  | 0.335 |  |  |  |
| item62 | 7.2 | 0.547 | 0.488 |  |  | 0.357 |  |  |  |
| item63 | 7.3 | 0.423 | 0.554 |  |  | 0.326 |  |  |  |
| item64 | 7.4 | 0.522 | 0.446 | 0.353 |  |  |  |  |  |
| item65 | 7.5 | 0.522 | 0.482 | 0.393 |  |  |  |  |  |
| item66 | 7.6 | 0.583 | 0.491 | 0.438 |  |  |  |  |  |
| item67 | 7.7 | 0.523 | 0.479 | 0.407 |  |  |  |  |  |
| item68 | 7.8 | 0.514 | 0.508 | 0.418 |  |  |  |  |  |
| item69 | 7.9 | 0.393 | 0.514 | 0.485 |  |  |  |  |  |
| item70 | 7.10 | 0.401 | 0.468 | 0.567 |  |  |  |  |  |
| **8-Review data present, notes and charts** | | | | | | | | | |
| item71 | 8.1 | 0.599 |  | 0.421 |  |  |  |  |  |
| item72 | 8.2 | 0.569 |  | 0.463 |  |  |  |  |  |
| item73 | 8.3 | 0.569 |  |  |  |  |  |  |  |
| item74 | 8.4 | 0.581 |  |  |  |  |  |  |  |

The boldface indicates salient (> 0.30) loading

**Supplementary Table (3): Exploratory factor analysis and factor loadings (EFA)**

|  |  | **After rotation Promax with Kaiser Normalization** | | | | | | | | **Communalities** |
| --- | --- | --- | --- | --- | --- | --- | --- | --- | --- | --- |
| **Item number** | **Section** | **Factor 1** | **Factor 2** | **Factor 3** | **Factor 4** | **Factor 5** | **Factor 6** | **Factor 7** | **Factor 8** |  |
| **1-Preparation for receiving patient** | | | | | | | | | |  |
| **1.1 Bed and the bed space preparation:** | | | | | | | | | |  |
| item1 | 1.1.1 |  |  |  |  | 0.348 | 0.907 |  |  | 0.873 |
| item2 | 1.1.2 |  |  |  |  |  | 0.898 |  |  | 0.837 |
| item3 | 1.1.3 |  |  |  |  |  | 0.808 |  |  | 0.727 |
| item4 | 1.1.4 |  |  | 0.306 |  | 0.366 | 0.821 |  |  | 0.746 |
| item5 | 1.1.5 |  |  |  |  |  | 0.906 |  |  | 0.844 |
| **1.2 Emergency equipment / machines preparation:** | | | | | | | | | |  |
| item6 | 1.2.1 |  | 0.363 | 0.348 |  | 0.801 | 0.414 |  |  | 0.749 |
| item7 | 1.2.2 |  | 0.318 |  |  | 0.727 | 0.432 |  |  | 0.724 |
| item8 | 1.2.3 |  | 0.307 | 0.302 |  | 0.755 | 0.488 |  |  | 0.789 |
| item9 | 1.2.4 |  |  | 0.391 |  | 0.702 | 0.404 |  |  | 0.665 |
| item10 | 1.2.5 |  |  | 0.307 |  | 0.645 |  |  |  | 0.552 |
| item11 | 1.2.5.1 |  | 0.344 | 0.305 | 0.366 | 0.835 |  |  |  | 0.799 |
| item12 | 1.2.5.2 |  |  | 0.329 |  | 0.620 | 0.337 | 0.301 |  | 0.623 |
| item13 | 1.2.6 |  |  |  |  | 0.422 |  |  |  | 0.214 |
| item14 | 1.2.6.1 |  |  |  |  | 0.438 |  |  |  | 0.249 |
| item15 | 1.2.7 |  |  |  | 0.369 | 0.537 | 0.428 | 0.431 |  | 0.595 |
| item16 | **1.3** | 0.300 |  |  |  |  | 0.328 | 0.706 |  | 0.600 |
| item17 | **1.4** |  |  |  |  |  |  | 0.810 |  | 0.743 |
| **2-Use recognized assessment framework to assess patient status, e.g. ABCDE approach** | | | | | | | | | |  |
| ***2.1 Airway:*** | | | | | | | | | |  |
| item18 | 2.1.1 |  |  | 0.300 |  |  |  | 0.843 | 0.344 | 0.820 |
| item19 | 2.1.2 |  |  |  |  |  |  | 0.763 | 0.337 | 0.676 |
| item20 | 2.1.3 |  |  |  | 0.317 |  |  | 0.734 | 0.356 | 0.671 |
| item21 | 2.1.4 |  |  |  | 0.326 |  |  | 0.354 | 0.803 | 0.777 |
| ***2.2 Breathing*** | | | | | | | | | |  |
| item22 | 2.2.1 |  |  |  |  |  |  | 0.340 | 0.790 | 0.740 |
| item23 | 2.2.2 |  |  |  | 0.472 |  |  | 0.466 | 0.713 | 0.785 |
| ***2.3 Circulation : Assess the patient’s circulation via*** | | | | | | | | | |  |
| item24 | 2.3.1 |  |  |  |  | -0.343 | 0.366 | 0.452 | 0.574 | 0.770 |
| item25 | 2.3.2 |  |  |  |  | -0.341 | 0.359 | 0.416 | 0.577 | 0.739 |
| item26 | 2.3.3 | 0.315 |  |  | 0.510 |  |  | 0.452 | 0.723 | 0.796 |
| item27 | 2.3.4 |  |  |  | 0.648 |  |  |  | 0.375 | 0.652 |
| item28 | 2.3.5 |  |  |  | 0.689 | 0.378 |  |  | 0.378 | 0.731 |
| item29 | 2.3.6 |  |  |  | 0.590 |  |  |  | 0.310 | 0.512 |
| ***2.4 Disability*** | | | | | | | | | |  |
| item30 | 2.4.1 |  |  |  |  |  | 0.339 | 0.536 |  | 0.344 |
| item31 | 2.4.2 |  | 0.321 |  |  |  |  | 0.321 |  | 0.275 |
| item32 | 2.4.3 | 0.392 |  |  | 0.363 |  | 0.527 | 0.361 |  | 0.456 |
| ***2.5 Exposure / environmental control*** | | | | | | | | | |  |
| item33 | 2.5.1 | 0.360 |  | 0.678 |  |  |  |  |  | 0.531 |
| item34 | 2.5.2 |  | 0.339 | 0.852 |  | 0.393 |  |  |  | 0.883 |
| item35 | ***2.6*** |  | 0.332 | 0.837 |  |  | 0.310 |  |  | 0.830 |
| item36 | ***2.7*** |  | 0.332 | 0.860 |  |  | 0.311 |  |  | 0.866 |
| **3-Measuring vital signs** | | | | | | | | | |  |
| item37 | 3.1 |  | 0.356 | 0.897 |  | 0.332 | 0.332 |  |  | 0.925 |
| item38 | 3.2 |  | 0.379 | 0.887 |  | 0.327 | 0.303 |  |  | 0.924 |
| item39 | 3.3 |  | 0.357 | 0.883 |  | 0.351 |  |  |  | 0.918 |
| item40 | 3.4 |  | 0.929 | 0.311 |  | 0.413 |  |  |  | 0.904 |
| item41 | 3.5 |  | 0.900 |  |  | 0.366 |  |  |  | 0.824 |
| item42 | 3.6 |  | 0.897 |  |  |  |  |  |  | 0.895 |
| item43 | 3.7 |  | 0.887 |  |  |  |  |  |  | 0.858 |
| item44 | 3.8 |  | 0.943 |  |  | 0.428 |  |  |  | 0.929 |
| item45 | 3.9 |  | 0.898 |  |  | 0.393 |  |  |  | 0.843 |
| item46 | 3.10 |  | 0.881 | 0.322 |  | 0.440 |  |  |  | 0.834 |
| **4-Wound/surgical site assessment** | | | | | | | | | |  |
| item47 | 4.1 |  | 0.683 | 0.629 |  | 0.395 | 0.318 |  |  | 0.816 |
| item48 | 4.2 |  | 0.596 | 0.550 |  | 0.376 | 0.447 |  |  | 0.739 |
| item49 | 4.3 |  | 0.518 | 0.403 | 0.303 |  | 0.437 |  |  | 0.576 |
| item50 | 4.4 | 0.392 | 0.806 |  |  |  |  | 0.428 |  | 0.809 |

**Supplementary Table (4): Exploratory factor analysis and factor loadings (EFA)**

|  |  | **After rotation Promax with Kaiser Normalization** | | | | | | | | **Communalities** |
| --- | --- | --- | --- | --- | --- | --- | --- | --- | --- | --- |
| **Item number** | **Section** | **Factor 1** | **Factor 2** | **Factor 3** | **Factor 4** | **Factor 5** | **Factor 6** | **Factor 7** | **Factor 8** |  |
| **5-Check fluid status** | | | | | | | | | |  |
| item51 | 5.1 | 0.458 |  | 0.338 | 0.878 |  |  | 0.519 |  | 0.850 |
| item52 | 5.2 | 0.488 |  |  | 0.852 |  |  | 0.558 |  | 0.876 |
| item53 | 5.3 | 0.437 |  | 0.357 | 0.855 |  |  | 0.516 |  | 0.855 |
| item54 | 5.4 | 0.803 |  |  | 0.525 |  |  | 0.472 |  | 0.790 |
| item55 | 5.5 | 0.609 |  | 0.638 |  |  |  |  |  | 0.802 |
| item56 | 5.6 | 0.635 |  | 0.502 |  |  |  |  |  | 0.709 |
| item57 | 5.7 | 0.588 |  | 0.639 |  |  |  |  |  | 0.793 |
| **6-Managing pain** | | | | | | | | | |  |
| item58 | 6.1 |  |  |  | 0.727 |  |  | 0.341 |  | 0.790 |
| item59 | 6.2 |  |  |  | 0.734 | 0.316 |  | 0.300 |  | 0.720 |
| item60 | 6.3 |  |  |  | 0.645 |  |  |  |  | 0.695 |
| **7-Safety** | | | | | | | | | |  |
| item61 | 7.1 | 0.828 |  | 0.347 | 0.440 |  |  | 0.309 |  | 0.714 |
| item62 | 7.2 | 0.732 |  | 0.403 | 0.476 |  |  | 0.380 |  | 0.789 |
| item63 | 7.3 | 0.816 |  |  | 0.318 |  |  |  |  | 0.885 |
| item64 | 7.4 | 0.814 |  |  | 0.372 |  |  |  |  | 0.717 |
| item65 | 7.5 | 0.818 |  |  | 0.373 |  |  |  |  | 0.784 |
| item66 | 7.6 | 0.878 |  |  | 0.416 |  |  |  |  | 0.718 |
| item67 | 7.7 | 0.795 |  |  | 0.449 |  |  |  |  | 0.792 |
| item68 | 7.8 | 0.827 |  |  | 0.427 |  |  |  |  | 0.704 |
| item69 | 7.9 | 0.769 |  |  | 0.380 |  |  |  |  | 0.697 |
| item70 | 7.10 | 0.813 |  |  | 0.321 |  |  |  |  | 0.576 |
| **8-Review data present, notes and charts** | | | | | | | | | |  |
| item71 | 8.1 | 0.505 | 0.415 |  | 0.719 |  |  |  | 0.505 | 0.609 |
| item72 | 8.2 | 0.442 | 0.495 |  | 0.671 |  |  |  | 0.442 | 0.660 |
| item73 | 8.3 | 0.418 |  |  | 0.430 |  | 0.335 | 0.406 | 0.430 | 0.707 |
| item74 | 8.4 | 0.406 |  |  | 0.483 |  |  | 0.354 | 0.406 | 0.710 |
| **Kaiser-Meyer-Olkin Measure of Sampling Adequacy = 0.666** | | | | | | | | | | |

The boldface indicates salient (> 0.30) loading

**Supplementary Figure (1):	CFA Confirmatory Factor Analysis by Structure Equation Modeling (n = 100)**

Model fit parameters CFI; IFI; RMSEA (1.000; 1.000; 0.081).

CFI = Comparative fit index; IFI = incremental fit index; and RMSEA = Root Mean Square Error of Approximation.

Model χ^2^; significance 1.751(0.001*)

**F1: Preparation for receiving patient**

**F2: Use recognized assessment framework to assess patient status, e.g. ABCDE approach**

**F3: Measuring vital signs**

**F4: Wound/surgical site assessment**

**F5: Check fluid status**

**F6: Managing pain**

**F7: Safety**

**F8: Review data present, notes and charts**

**Supplementary Table (5): Corrected Item-Total Correlations**

| **Item number** | **Section** | **Correlation** |
| --- | --- | --- |
| **1-Preparation for receiving patient** | | |
| **1.1 Bed and the bed space preparation:** | | |
| item1 | 1.1.1 | 0.765* |
| item2 | 1.1.2 | 0.708* |
| item3 | 1.1.3 | 0.663* |
| item4 | 1.1.4 | 0.746* |
| item5 | 1.1.5 | 0.740* |
| **1.2 Emergency equipment / machines preparation:** | | |
| item6 | 1.2.1 | 0.770* |
| item7 | 1.2.2 | 0.734* |
| item8 | 1.2.3 | 0.785* |
| item9 | 1.2.4 | 0.712* |
| item10 | 1.2.5 | 0.456* |
| item11 | 1.2.5.1 | 0.670* |
| item12 | 1.2.5.2 | 0.619* |
| item13 | 1.2.6 | 0.848* |
| item14 | 1.2.6.1 | 0.800* |
| item15 | 1.2.7 | 0.734* |
| item16 | **1.3** | 0.770* |
| item17 | **1.4** | 0.734* |
| **2-Use recognized assessment framework to assess patient status, e.g. ABCDE approach** | | |
| ***2.1 Airway:*** |  |  |
| item18 | 2.1.1 | 0.467* |
| item19 | 2.1.2 | 0.544* |
| item20 | 2.1.3 | 0.679* |
| item21 | 2.1.4 | 0.723* |
| ***2.2 Breathing*** |  |  |
| item22 | 2.2.1 | 0.626* |
| item23 | 2.2.2 | 0.630* |
| ***2.3 Circulation : Assess the patient’s circulation via*** | | |
| item24 | 2.3.1 | 0.729* |
| item25 | 2.3.2 | 0.703* |
| item26 | 2.3.3 | 0.822* |
| item27 | 2.3.4 | 0.617* |
| item28 | 2.3.5 | 0.606* |
| item29 | 2.3.6 | 0.825* |
| ***2.4 Disability*** |  |  |
| item30 | 2.4.1 | 0.404* |
| item31 | 2.4.2 | 0.445* |
| item32 | 2.4.3 | 0.404* |
| ***2.5 Exposure / environmental control*** | | |
| item33 | 2.5.1 | 0.521* |
| item34 | 2.5.2 | 0.427* |
| item35 | ***2.6*** | 0.547* |
| item36 | ***2.7*** | 0.526* |
| **3-Measuring vital signs** |  |  |
| item37 | 3.1 | 0.855* |
| item38 | 3.2 | 0.834* |
| item39 | 3.3 | 0.843* |
| item40 | 3.4 | 0.877* |
| item41 | 3.5 | 0.886* |
| item42 | 3.6 | 0.871* |
| item43 | 3.7 | 0.806* |
| item44 | 3.8 | 0.721* |
| item45 | 3.9 | 0.697* |
| item46 | 3.10 | 0.681* |
| **4-Wound/surgical site assessment** |  |  |
| item47 | 4.1 | 0.960* |
| item48 | 4.2 | 0.941* |
| item49 | 4.3 | 0.947* |
| item50 | 4.4 | 0.856* |

**Supplementary Table (6): Corrected Item-Total Correlations**

| **Item number** | **Section** | **Correlation** |
| --- | --- | --- |
| **5-Check fluid status** |  |  |
| item51 | 5.1 | 0.500* |
| item52 | 5.2 | 0.609* |
| item53 | 5.3 | 0.853* |
| item54 | 5.4 | 0.897* |
| item55 | 5.5 | 0.856* |
| item56 | 5.6 | 0.859* |
| item57 | 5.7 | 0.642* |
| **6-Managing pain** |  |  |
| item58 | 6.1 | 0.940* |
| item59 | 6.2 | 0.920* |
| item60 | 6.3 | 0.956* |
| **7-Safety** |  |  |
| item61 | 7.1 | 0.796* |
| item62 | 7.2 | 0.689* |
| item63 | 7.3 | 0.814* |
| item64 | 7.4 | 0.835* |
| item65 | 7.5 | 0.861* |
| item66 | 7.6 | 0.925* |
| item67 | 7.7 | 0.834* |
| item68 | 7.8 | 0.881* |
| item69 | 7.9 | 0.827* |
| item70 | 7.10 | 0.842* |
| **8-Review data present, notes and charts** | | |
| item71 | 8.1 | 0.846* |
| item72 | 8.2 | 0.836* |
| item73 | 8.3 | 0.775* |
| item74 | 8.4 | 0.834* |

Correlation: Pearson coefficient	*: Statistically significant at p ≤ 0.05

**Supplementary Table (7): Correlations Between dimensions**

|  | | **1-Preparation for receiving patient** | **2-Use recognized assessment framework to assess patient status, e.g. ABCDE approach** | **3-Measuring vital signs** | **4-Wound/surgical site assessment** | **5-Check fluid status** | **6-Managing pain** | **7-Safety** | **8-Review data present, notes and charts** | **Overall** |
| --- | --- | --- | --- | --- | --- | --- | --- | --- | --- | --- |
| **1-Preparation for receiving patient** | **r** |  |  |  |  |  |  |  |  |  |
|  | **p** |  |  |  |  |  |  |  |  |  |
| **2-Use recognized assessment framework to assess patient status** | **r** | 0.220* |  |  |  |  |  |  |  |  |
|  | **p** | 0.028* |  |  |  |  |  |  |  |  |
| **3-Measuring vital signs** | **r** | 0.444* | 0.243* |  |  |  |  |  |  |  |
|  | **p** | <0.001* | 0.015* |  |  |  |  |  |  |  |
| **4-Wound/surgical site assessment** | **r** | 0.443* | 0.205* | 0.806* |  |  |  |  |  |  |
|  | **p** | <0.001* | 0.047* | <0.001* |  |  |  |  |  |  |
| **5-Check fluid status** | **r** | 0.293* | 0.495* | 0.451* | 0.282* |  |  |  |  |  |
|  | **p** | 0.003* | <0.001* | <0.001* | 0.004* |  |  |  |  |  |
| **6-Managing pain** | **r** | 0.214* | 0.223* | 0.223* | 0.254* | 0.369* |  |  |  |  |
|  | **p** | 0.033* | 0.026* | 0.026* | 0.011* | <0.001* |  |  |  |  |
| **7-Safety** | **r** | 0.231* | 0.413* | 0.225* | 0.234* | 0.440* | 0.506* |  |  |  |
|  | **p** | 0.021* | <0.001* | 0.024* | 0.019* | <0.001* | <0.001* |  |  |  |
| **8-Review data present, notes and charts** | **r** | 0.277* | 0.482* | 0.325* | 0.231* | 0.610* | 0.220* | 0.562* |  |  |
|  | **p** | 0.005* | <0.001* | 0.001* | 0.021* | <0.001* | 0.028* | <0.001* |  |  |
| **Overall competence** | **r** | 0.630* | 0.690* | 0.675* | 0.488* | 0.743* | 0.443* | 0.610* | 0.696* |  |
|  | **p** | <0.001* | <0.001* | <0.001* | <0.001* | <0.001* | <0.001* | <0.001* | <0.001* |  |

r: Pearson coefficient	*: Statistically significant at p ≤ 0.05

**Supplementary Table (8): Cronbach's Alpha**

|  | **N of Items** | **Cronbach’s alpha** |
| --- | --- | --- |
| 1-Preparation for receiving patient | 17 | 0.903 |
| 2-Use recognized assessment framework to assess patient status, e.g. ABCDE approach | 19 | 0.890 |
| 3-Measuring vital signs | 10 | 0.900 |
| 4-Wound/surgical site assessment | 4 | 0.945 |
| 5-Check fluid status | 7 | 0.859 |
| 6-Managing pain | 3 | 0.932 |
| 7-Safety | 10 | 0.905 |
| 8-Review data present, notes and charts | 4 | 0.841 |
| **Overall** | **74** | **0.919** |
